# *Ataque de nervios*: The impact of sociodemographic, health history, and psychological dimensions on Puerto Rican adults

**DOI:** 10.3389/fpsyt.2023.1013314

**Published:** 2023-01-20

**Authors:** Marcos I. Roche-Miranda, Alisha M. Subervi-Vázquez, Karen G. Martinez

**Affiliations:** Department of Psychiatry, Medical Sciences Campus, University of Puerto Rico, San Juan, Puerto Rico

**Keywords:** *ataque de nervios*, anxiety, affect, personality, sociodemographic, trauma, Puerto Rico, health history

## Abstract

**Introduction:**

*Ataque de nervios* (ADN) is a cultural syndrome prevalent in Puerto Ricans characterized as an episode of intense emotional upset due to overwhelming stress.

**Methods:**

The *Ataque de Nervios Questionnaire*, developed at the Center for the Study and Treatment for Fear and Anxiety (CETMA), served as the diagnostic tool for this retrospective secondary data analysis. We evaluated three models regarding ADN’s function as a marker of (1) sociodemographic vulnerability, (2) health history risk, and (3) psychological vulnerability. This last model was subdivided to assess the scores of screening tests regarding anxiety (Anxiety Sensitivity Inventory, Beck Anxiety Inventory, and State-Trait Anxiety Inventory), affect (Beck Depression Inventory, Emotional Dysregulation Scale, Positive and Negative Affective Schedule), personality (NEO Five-Factor Inventory), and trauma (considering the responses to the Childhood Trauma Questionnaire and the Life Event Checklist).

**Results:**

Our study sample had a total of 121 Puerto Rican adult patients from CETMA out of which 75% had ADN. We differentiated subjects according to their ADN status with t-tests and Mann-Whitney U tests and evaluated our models using logistic regressions. People with ADN showed more anxiety, depressive symptoms, emotional dysregulation, and negative affect than those without ADN. They also revealed lower positive affect and agreeableness. Highly extraverted but minimally agreeable personalities related to ADN. Living with a partner and being employed were risk factors for ADN. Having higher educational levels showed the strongest effect size: it greatly reduced the odds of an *ataque*.

**Discussion:**

These characteristics suggest a distinct profile of ADN seen in employed, educated, adult Puerto Ricans living on the Island experiencing anxiety. Our study provides clinical tools to comprehend our patients’ ADN experience, enriching our practice as culturally competent health providers.

## 1. Introduction

*Ataque de nervios* (ADN) is a cultural syndrome. Cultural syndromes are one of the three cultural concepts of distress described in the Diagnostic and Statistical Manual of Mental Disorders, Fifth Edition, Text Revision (DSM-5-TR). The DSM-5-TR explains cultural concepts of distress as how individuals “experience, understand, and communicate suffering, behavioral problems, or troubling thoughts and emotions” (1). They may exist alone or coexist with other psychiatric disorders, potentially influencing the “clinical presentation, course, and outcome.” Guarnaccia et al. (2) proposed that *nervios* was more than a diffuse idiom of distress; they suggested that these different ways in which distress may be experienced and expressed by Puerto Ricans could signal different sources of suffering (2, 3). They coined this schema “popular nosology of suffering.” ADN affects 7–15% of Latino adults in their lifetime (4) and 15% of Puerto Ricans (4–6). ADN is described as an episode of intense emotional upset (e.g., anxiety, anger, or grief) due to a stressful event where the person typically describes a sense of being out of control (1, 4, 5). Symptoms reported in the DSM-5-TR include trembling, uncontrollable screaming or crying, aggressive or suicidal behavior, and depersonalization or derealization. Symptoms may be experienced for only a few minutes or longer. Some patients experience a *trigger* while others have no identifiable cause for these episodes. Usually, the event is related to the family, such as news of the death of a close relative, conflicts with a spouse or children, or witnessing an accident involving a family member. After the *episode*, people often experience amnesia from what occurred and rapidly return to their usual level of functioning. ADN is a culturally normative experience in response to overwhelming stress, where some report feeling better after an episode and may not express fear of having subsequent attacks. This may be one reason some do not seek treatment for the episodes (7–9). *Emotional* expressions of stressful situations vary across ethnicities and cultures (10–12). In Puerto Ricans, *physiological* expressions of threats are more common than anxious thoughts. These physiological expressions were identified in various studies on self-identified Puerto Ricans (13–17). Due to its variability in experience and expression, ADN has been studied from many perspectives to understand it in greater depth. Researchers have studied ADN’s association with psychiatric disorders like panic attacks, panic disorder, dissociative disorders, intermittent explosive disorder, anxiety disorders, and other trauma- and stressor-related disorders (1). ADN is orthogonally distinguished from panic attacks with the latter having “(1) a discrete period of intense fear or discomfort, (2) the presence of four or more out of the DSM-5-TR list of 13 panic symptoms, and (3) a crescendo occurring within 10 min” (8). However, some *ataques* can meet the criteria for panic (either attack or disorder) while other *ataques* may not. Even so, many Puerto Ricans consider most of their panic attacks to be *ataques*. Panic attacks can occur in response to a stressor or uncued. ADN and panic disorders can both occur in response to a stressful event, even though the latter occasionally occur unexpectedly. Popular distress categories, like ADN, do not have established criteria like DSM disorders (e.g., panic disorder) which have specified symptoms, recurrence patterns, and time frames. This variability in the relationship between cultural concepts of distress and DSM diagnoses makes ADN identification and treatment particularly challenging.

To provide culturally competent care, it is imperative we comprehend ADN through a biopsychosocial perspective. At the Center for the Study and Treatment of Fear and Anxiety (CETMA) at the University of Puerto Rico, Medical Sciences Campus (UPR-MSC), an *Ataque de Nervios Questionnaire* (ADNQ) was designed to screen and assess the expressions of these *ataques*. The first question screens for having experienced an ADN, while questions two through eleven address the expressions of these *ataques*. The *purpose* of this study is to compare the biopsychosocial factors between and amongst subjects with and without ADN and evaluate the factors’ associations with ADN. In this paper, we grouped these factors into three models: the Sociodemographic Vulnerability model, the Health History Risk model, and the Psychological Vulnerability model.

### 1.1. Sociodemographic Vulnerability model

*Ataque de nervios* (ADN) has been studied regarding its association with age, gender, education level, employment status, and if the person is living with a partner. One study which presented data from a 1987 psychiatric epidemiological survey conducted in adults aged 17 to 68 living in Puerto Rico (18, 19) found that women over the age of 45, who had less than a high school education, who were not working, and who were widowed, separated, or divorced were more likely to experience an *ataque* (6). Between 2002 and 2003, the National Latino and Asian American Study (NLAAS) –one of the largest population-based surveys conducted in the United States with this sample– evaluated adults who were Latino, Hispanic, or of Spanish origin (20). Using this study’s results, Guarnaccia et al. (4) identified the same trend of increased ADN reports from women with the mentioned marital status (4). However, they did not find associations between age or education with ADN. This same study proposed that ADN served as a *marker of social and psychiatric vulnerability* in the Latino population. Social vulnerability is defined as those contextual factors (e.g., disparities in gender relations; racial discrimination; and political and economic circumstances, including poverty) that differentially and adversely impact populations (21). Latino individuals who are significantly affected by these *social vulnerability factors* are at greater risk of “mental health problems and related disability” (4). Due to these differences in results among the literature, we chose to analyze these sociodemographic factors in our Puerto Rican sample.

### 1.2. Health History Risk model

*Ataque de nervios* (ADN) has been frequently described in patients with other DSM diagnoses. One study, which evaluated 303 participants from a primary care sample of Puerto Ricans aged 50 years and older living in the mainland United States found that 84% of participants with a lifetime *ataque* event met the criteria for at least one anxiety, mood, suicidal, psychotic, or substance use problem (22). ADN has even been determined to be the strongest and most consistent predictor of having another mental health disorder (4). In the mentioned study, those who reported an *ataque* were more likely to have used general medical and specialty mental health services and to have been hospitalized for a mental health problem. Additionally, 80% met the criteria for any lifetime psychiatric disorder and were five times more likely to have suicidal symptoms, twice as likely to have psychotic symptoms, and five times more likely to have depression or anxiety in comparison with the total sample of Latinos (4). Other psychiatric diagnoses associated with ADN are dysthymia, agoraphobia, phobic disorder, substance use disorder, post-traumatic stress disorder, and panic disorder (4, 6, 23–28). Another study identified that Puerto Ricans reported more somatic symptoms, using the results from the Hispanic Health and Nutrition Examination Survey (HHANES) (13). The HHANES was conducted between 1982 and 1984 among a sample of 7,462 Mexican Americans in the five southwestern states, 2,834 Puerto Ricans in the New York area, and 1,357 Cubans in Miami, Florida aged from 6 months to 74 years (29). Consistent with the findings of the study that used the HHANES, Puerto Ricans have shown increased physiological responses to stimuli compared to White non-Hispanics (14). These somatic responses are expressed in ADN. It has been hypothesized that since the manifestation of psychological symptoms can carry a greater stigma in collectivistic societies, racial/ethnic minorities are more likely to deny them and somaticize distress to avoid discrimination (30). This somatization can lead them to seek medical care instead of mental healthcare (31). In our study, we include an analysis of family physical and mental health diagnoses because they could reveal biological generational effects surrounding this cultural syndrome (32). Therefore, we considered the evaluation of these factors important for our comprehension of ADN as a marker of an individual’s health history risk.

### 1.3. Psychological Vulnerability model

*Ataque de nervios* (ADN) has shown a link with anxiety (33–35), depression (36), emotional dysregulation (37), and affect (33). A 2005 study, which included 177 participants drawn from a previous work of 275 Puerto Ricans residing in the Puerto Rico metropolitan area (38), identified that patients with ADN reported higher anxiety sensitivity (35). Additionally, patients who had an ADN within a month of screening had higher *Anxiety Sensitivity Index* scores (33). *Affect regulation*, on the other hand, refers to the ability to recognize and evaluate our emotions, facilitating socially acceptable management and responses (39–43). Its absence, *emotional dysregulation*, can be more severe in the presence of anxiety (15, 44) and has been linked with childhood trauma (26, 37, 45, 46). ADN is frequently associated with a history of traumatic events (15, 34, 37, 45, 47–49). A study performed among 2,951 Puerto Rican children and adolescents aged 5 to 13 years-old residing in South Bronx, New York and the Standard Metropolitan Areas in Puerto Rico showed that children with *ataques* had a higher lifetime exposure to violence and trauma (7). They suggested that violence could culturally shape a child’s expression of distress and increase the risk for future psychopathology development. Researchers hypothesized that Hispanic women with co-occurring psychiatric diagnoses would be at greater risk for other social vulnerabilities (e.g., community violence and criminal justice involvement), even when controlling for socioeconomic resources (21). This study was conducted at nine sites within the United States, examined data from 2,534 women enrolled in the Women, Co-occuring Disorders and Violence Study, and included Hispanics, non-Hispanic Blacks/African Americans, and non-Hispanic Whites. Additionally, Hispanic women with trauma histories were more likely to have more severe mental health disorders (21, 50) as per the previous study and another work which evaluated data from the 1990–1992 National Comorbidity Survey (NCS) (51). The NCS provided information on the prevalence of DSM-III-R disorders from a sample of 8,098 English-speaking respondents in the United States aged 15–54 years. Lastly, personality factors such as high neuroticism (15) and low extraversion have been described as risk factors for mood disorders (52–54) and should be considered when treating Latino patients (15). These relationships led to the characterization and analysis of our Psychological Vulnerability model.

The objective of this study was to understand how biopsychosocial circumstances influence the ADN experience. We also wanted to evaluate the clinical utility of these factors in the identification of ADN. With the associations revealed through the proposed models, our study will facilitate an efficient screening of ADN. We hypothesize that sociodemographic factors related to ADN will be advanced adult age, being female, having a low education, being unemployed, and not living with a partner. Regarding health history factors, we hypothesized ADN will be related to both personal and family history of other DSM diagnoses. For the Psychological Vulnerability model, we hypothesized ADN will be related to increased anxiety sensitivity, affect dysregulation, high neuroticism and low extraversion, and a history of traumatic life events. To provide culturally competent care in Puerto Rico, it is imperative we comprehend how ADN relates to the biopsychosocial context of our patients. Our models will serve as a clinically relevant tool for early identification of risk and individualized patient management according to our patients’ ADN experience.

## 2. Materials and methods

### 2.1. Participants and inclusion criteria

This is a secondary data analysis study using an observational, retrospective medical record review design on a Puerto Rican sample. We included data from all adult (18 years or older) patient CETMA medical records from 2014 to 2019 that responded to the first item in the ADNQ. The initial interview at CETMA was designed, for research purposes, based on the Structured Clinical Interview for DSM Disorders, so it includes DSM-5 criteria for each diagnostic screening. CETMA is a clinical and research center ascribed to the Department of Psychiatry of the UPR-MSC. It receives psychiatry residents, psychology interns, and medical students. Since residents and psychology interns rotate at the clinic, some have participated in patient interviews. However, a psychiatrist and a psychologist supervise all evaluations, increasing diagnostic accuracy and reliability. CETMA is in San Juan, Puerto Rico, but serves patients from all over the Island.

Our study sample had a total of 121 adults out of which 90 (75%) had ADN. The average age was 36 (*SD* = 12.39). Most were women (64%), had an education level of college or above (79%), were employed (67%), did not live with a partner (63%), and answered the Life Event Checklist (LEC-5) (63%). Most people had a physical (58%) or other DSM (84%) diagnosis. The presence of family physical (82%) or mental health (66%) diagnoses were also common.

The University of Pittsburgh’s Human Research Protections Office Retrospective Medical Record Review served as a base for the design’s development (55). There was no direct contact with participants and no subjects’ identifiers were used; therefore, no consents were necessary. The study was approved by the University of Puerto Rico Human Research Subjects Protection Office as protocol A3910119.

### 2.2. Data collection and variables

**FIGURE 1:**
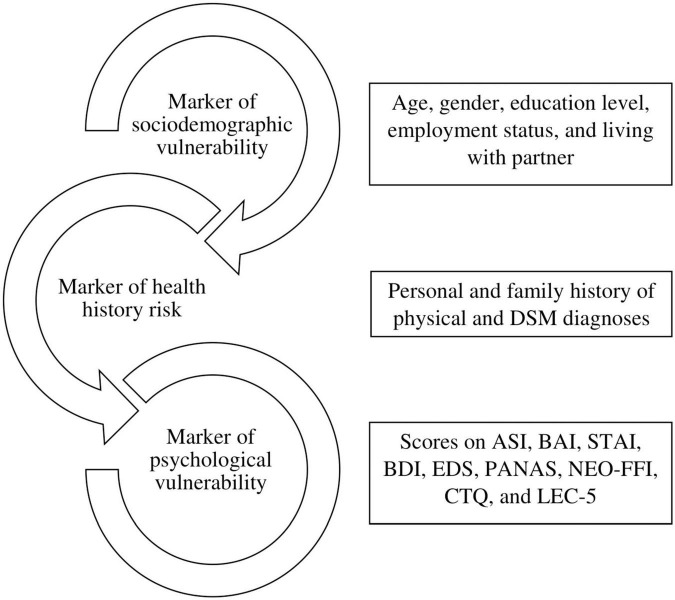
Proposed models on how *ataque de nervios* can be used clinically. Abbreviations: DSM, Diagnostic and Statistical Manual of Mental Disorders; ASI, Anxiety Sensitivity Index; BAI, Beck Anxiety Inventory; STAI, State-Trait Anxiety Inventory; BDI, Beck Depression Inventory; EDS, Emotional Dysregulation Scale; PANAS, Positive and Negative Affective Schedule; NEO-FFI, NEO Five-Factor Inventory; CTQ, Childhood Trauma Questionnaire; LEC-5, Life Event Checklist.

For this study, we collected the following data from the medical records: sociodemographic data, psychiatric diagnoses, medical history, and family medical history. A clinician using DSM-5 criteria determined the patients’ psychiatric diagnoses. Medical history and family medical history were self-reported. We retrieved the answers for the LEC-5, the first item of the ADNQ, and the scores of eight psychological screening tests. The variables were grouped into three clinical models, which guided our analyses. Each model refers to how ADN can be used as a clinical marker (Figure 1).

#### 2.2.1. *Ataque de Nervios Questionnaire*

We identified ADN based on the previous literature (4, 56) that cases with ADN are those that answer “Yes” to the question “Have you ever had an episode or *ataque de nervios* in which you felt out of control?” This is the current standard for diagnosing ADN based on the DSM-5 definition. The ADNQ developed at CETMA is composed of eleven questions, the first question screens for having experienced an ADN, while questions two through eleven address the expressions of these *ataques*, frequency of *ataques*, precipitating factors, the impact of the episodes, symptom management, as well as the name that was given to the event.

#### 2.2.2. Variables to evaluate the Sociodemographic Vulnerability model

Sociodemographic variables included age, gender, education level, employment, and living with a partner. Besides age, all other independent variables were considered dichotomous. Gender was categorized as Men or Women; the Clinic’s patients reported no other genders. The levels of education were: High school or below or College or above. In the Living with partner variable, those patients that were either married or lived with a significant other were included. The subjects that were divorced or widowed were grouped in the Not living with partner category.

#### 2.2.3. Variables to evaluate the Health History Risk model

Clinical independent variables include a history of physical medical diagnosis, history of other DSM-5 diagnoses, family physical health history, and family mental health history. The clinical variables were categorized as Yes or No (referring to presence or absence, respectively).

#### 2.2.4. Variables to evaluate the Psychological Vulnerability model

This model was subdivided into four categories. For the *anxiety* category, we used the scores from the Anxiety Sensitivity Inventory (ASI), the Beck Anxiety Inventory (BAI), and the State-Trait Anxiety Inventory (STAI). For the *affect* category, we used the scores from the Beck Depression Inventory (BDI), Emotional Dysregulation Scale (EDS), and Positive and Negative Affective Schedule (PANAS). The *personality* category used the scores from the NEO Five-Factor Inventory (NEO-FFI). Lastly, our *trauma* category used the scores from the components of the Childhood Trauma Questionnaire (CTQ) and if the person answered the Life Event Checklist (LEC-5).

##### 2.2.4.1. Anxiety Sensitivity Inventory

The ASI is composed of 16 items and is used to evaluate the fear related to anxiety symptoms due to them being perceived as harmful. Each item is rated on a five-point Likert scale ranging from 0 (very little) to 4 (very much). It has been shown that the ASI has good internal consistency, with alpha coefficients 0.79–0.90 (57). This index was validated in Spanish using a Spanish clinical sample (58).

##### 2.2.4.2. Beck Anxiety Inventory

The Beck Anxiety Inventory (BAI) has 21 items, and it evaluates the severity of anxiety. BAI items are rated on a four-point scale: 0 (not at all) to 3 (severely). The English version of the BAI has an internal consistency of α = 0.92 (59). A study that used the Spanish version of the test including the Spanish general population obtained an alpha coefficient of 0.93 (60). In another study using elderly Puerto Rican participants, the BAI demonstrated high internal consistency, α = 0.95 (61).

##### 2.2.4.3. State-Trait Anxiety Inventory

The STAI consists of 20 items to measure the temporary condition of state anxiety and 20 items to measure the longstanding quality of trait anxiety (62). The four-point scale for state anxiety ranges from 1 (not at all) to 4 (very much so), while the trait anxiety scale ranges from 1 (almost never) to 4 (almost always). The internal consistency of the Spanish version of this test, which included a Puerto Rican sample, ranges from 0.82 to 0.95, according to Spielberger, Gonzalez-Reigosa, Martinez-Urrutia, Natalicio, and Natalicio (63). This inventory has been used in several studies that have included Puerto Ricans (64). This inventory has been validated for the Puerto Rican population studied by Virella, Arbona, and Novy (65).

##### 2.2.4.4. Beck Depression Inventory

The BDI-II is a 21-item multiple-choice self-report inventory. The BDI is a widely used instrument that measures the existence and severity of depression. Item scores range from 0 to 3. Alpha internal consistency coefficients for the scale have ranged from 0.88 to 0.93 (66, 67). In a sample of college-aged Puerto Rican individuals, an adapted version of the BDI (BDI-S) showed high internal consistency (α = 0.88) (68).

##### 2.2.4.5. Emotional Dysregulation Scale

The EDS consists of 40 items that evaluate emotional changes and the inability to manage emotions. The Spanish version of the EDS has an internal consistency of α = 0.97 in the Puerto Rican population studied (64).

##### 2.2.4.6. Positive and Negative Affective Schedule

The PANAS evaluates the experience of emotions, regarding positive (e.g., excitement) and negative (e.g., fear) affect and is composed of 10-item negative emotional scales and 10 positive emotional scales. Participants are asked to rate the extent to which they have experienced specific emotions using a five-point Likert scale. The PANAS was adapted for the Spanish-speaking population, showing elevated internal consistency ranging from 0.87 to 0.91, considering affect dimensions (69).

##### 2.2.4.7. NEO Five-Factor Inventory

The NEO Five-Factor Inventory (NEO-FFI) has 60 items that evaluate personality traits in five different dimensions (12 items each): neuroticism, extraversion, openness, agreeableness, and conscientiousness. Items are scored following a five-point Likert scale from 1 (strongly disagree) to 5 (strongly agree). Even though this inventory has not been validated for the Puerto Rican population, the long version has shown high internal consistency for this population, α = 0.95 (70).

##### 2.2.4.8. Childhood Trauma Questionnaire

The Childhood Trauma Questionnaire (Spanish Version-CTQ) (71) is a 28-item self-report scale designed for children over 12 years old to adults, which retrospectively assesses traumatic experiences during childhood. The instrument measures 5 types of maltreatment: sexual, physical, and emotional abuse; sexual and physical neglect. Responses are on a 5-point Likert scale, ranging from “never true” 0 to “very often true” 4 (72). Item examples include: “People in my family called me things like stupid,” and “I believe I was sexually abused.” A score higher than 5 in each subscale indicates the presence of a traumatic experience, and although scores vary across scales, 10 or more is considered a moderate to severe traumatic experience. The CTQs psychometric properties have been documented, including alpha internal reliability for each scale ranging from 0.62 to 0.92 (72) and validated (73, 74).

##### 2.2.4.9. Life Event Checklist

The Life Event Checklist (LEC-5) is a 25-item self-report measure (75, 76) that examines experiences with potentially traumatic events such as crime, general disaster, and sexual and physical assault using a yes/no format. For each event endorsed, respondents are asked to identify if they directly experienced the event or observed it. The psychometric properties for its Spanish version are unavailable and the scale lacks validation for the Puerto Rican population.

### 2.3. Statistical analysis

The analytical plan was established to first evaluate if there were differences between all variables in subjects with ADN and those without ADN using *t*-tests. If D’Agostino-Pearson goodness-of-fit measures identified variables with non-normal distributions, we used non-parametric Mann–Whitney *U* tests instead.

Logistic regression analyses evaluated the proposed models with ADN diagnosis as the dependent variable and a cutoff point of 0.5. Before the regressions, a correlation matrix for the 30 variables (29 independent variables) was checked for collinearity with a threshold of 0.70. We compared the models based on their Akaike information criterion (AIC); the lowest score signifies the best. The difference in AIC between the best model and the others was calculated to see if they were comparable, according to the recommendations of Burnham and Anderson (77). They outline a rule of thumb where AIC differences of 2 or less mean a substantial level of empirical support for the model, a difference ranging from 4 to 7 is considerably less support and differences greater than 10 mean essentially no support for the model.

The statistical analyses were made through Microsoft Excel for Microsoft 365 MSO (16.0.14228.20216) 64-bit, with support from the Analysis ToolPak and Charles Zaointz’s (78) Real Statistics Resource Pack software (Release 7.6), Copyright (2013–2022). For all *p*-values, the significance α = 0.05.

In this paper, we explore the relationship between experiencing an ADN to various sociodemographic factors which have been previously associated with ADN in Puerto Rico or in the USA. These factors include age, gender, education level, employment status, and if the person is living with a partner. We also examine the relationship between experiencing an ADN to physical diagnosis, other DSM diagnoses, and a family history of physical and mental diagnoses, categorized as health history factors. Lastly, we explore the relationship between experiencing an ADN to psychological vulnerability factors, particularly anxiety, affect, personality, and trauma. The goal of these analyses is to assess how each dimension impacts ADN.

### 2.4. Missing values

This study’s sample had an overall missing data of 24.1%. Various categories contained differing amounts of missing data, but PANAS Positive and LEC-5 had the most (48.76% for both). We chose multiple imputations over pairwise deletion to manage the missing data without losing power or introducing bias. The method was the fully conditional specification approach with 10 iterations, using ADN diagnosis as the outcome variable during the imputations. The pooled mean of the iterations’ datasets became our study sample. The pre-imputation dataset was comparable to the post-imputation dataset; they both presented a homogeneous sample.

## 3. Results

After allocating subjects according to their ADN status, the tests of independence in terms of sociodemographic variables, including the LEC-5, and their medical and family history, showed no statistically significant differences in their distributions. Table 1 presents the descriptive statistics of the study sample.

**TABLE 1:** Descriptive statistics of the post-imputation study sample (*N* = 121), with and without *ataque de nervios*, from a specialized anxiety clinic in Puerto Rico.

	ADN, *n* = 91 (75%)	No ADN, *n* = 30 (25%)
**Sociodemographic**		
Age (Mean ± SD)	35.30 ± 12.21	37.50 ± 13.03
Gender		
Men	29 (32%)	14 (47%)
Women	62 (68%)	16 (53%)
Education		
College and above	69 (76%)	27 (90%)
High school and below	22 (24%)	3 (10%)
Employment		
Employed	61 (67%)	20 (67%)
Unemployed	30 (33%)	10 (33%)
Living with partner?		
Yes	35 (38%)	10 (33%)
No	56 (62%)	20 (67%)
**Health history**		
Physical diagnoses		
Yes	53 (58%)	17 (57%)
No	38 (42%)	13 (43%)
Other DSM diagnoses		
Yes	74 (61%)	28 (93%)
No	17 (39%)	2 (7%)
Family physical diagnoses		
Yes	74 (61%)	25 (83%)
No	17 (39%)	5 (17%)
Family mental health diagnoses		
Yes	62 (68%)	18 (60%)
No	29 (32%)	12 (40%)
**Trauma**		
LEC-5		
Yes	59 (65%)	17 (57%)
No	32 (35%)	13 (43%)

There were no statistically significant differences between the groups. Abbreviation: SD, standard deviation; DSM, Diagnostic and Statistical Manual of Mental Disorders; LEC-5, Life Event Checklist.

### 3.1. Mann–Whitney *U* tests and *t*-tests

Table 2 summarizes the results of the Mann–Whitney *U* tests and *t*-tests. Statistically significant differences were found regarding all anxiety severity measures. ASI scores of people with ADN (Mdn = 45) were higher than the scores of those without ADN (Mdn = 35.5), *U* = 910, *z* = 2.73, *p* = 0.006. BAI scores of people with ADN (Mdn = 23) almost doubled the scores of people without ADN (Mdn = 12), *U* = 853, *z* = 3.07, *p* = 0.002. State Anxiety was higher in people with ADN (*M* = 43.53, *SD* = 10.22) than without ADN (*M* = 39.83, *SD* = 8.03), *t*(62.51) = 2.03, *p* = 0.05. The trend is the same with Trait Anxiety, in that people with ADN (Mdn = 53) had higher scores than people without ADN (Mdn = 39.5), *U* = 646, *z* = 4.31, *p* < 0.001.

**TABLE 2:** Statistical analyses relating psychological test scores with the presence of *ataque de nervios*.

	Median ADN	Median No ADN	Mean (SD) ADN	Mean (SD) No ADN	Mann–Whitney *U* tests U	*t*-tests t
**Anxiety**						
ASI	45	35.5			910**	
BAI	23	12			853**	
State anxiety			43.53 (10.22)	39.83 (8.03)		2.03*
Trait anxiety	53	39.5			646***	
**Affect**						
BDI	20	11			867.5**	
EDS	134	88			825.5***	
PANAS (+)			29.76 (7.76)	33 (7.40)		2.05*
PANAS (−)			30.03 (8.46)	25.1 (7.19)		3.11**
**Personality**						
NEO-FFI						
Neuroticism	64	60.5			1136.5	
Extraversion			47.07 (11.65)	45.6 (12.58)		0.59
Openness			55.67 (11.32)	52.8 (13.73)		1.14
Agreeableness			43.77 (11.22)	54.83 (10.52)		4.91***
Conscientiousness			43.58 (10.63)	42.07 (9.76)		0.72
**Trauma**						
CTQ						
Emotional abuse			10.73 (4.58)	8.97 (4.28)		1.92
Physical abuse	7	7			1356.5	
Sexual abuse	5	5			1181	
Emotional neglect			11.82 (4.54)	10.57 (5.51)		1.25
Physical neglect	7	6			1291	
Minimization/denial			8.37 (2.78)	8.83 (3.37)		0.74

Abbreviations: SD, standard deviation; ASI, Anxiety Sensitivity Index; BAI, Beck Anxiety Inventory; BDI, Beck Depression Inventory; EDS, Emotional Dysregulation Scale; PANAS, Positive and Negative Affective Schedule; NEO-FFI, NEO Five-Factor Inventory; CTQ, Childhood Trauma Questionnaire. * *p* ≤ 0.05, ** *p* ≤ 0.01, *** *p* ≤ 0.001.

All affect regulation tests yielded statistically significant results. BDI scores for people with ADN (Mdn = 20) nearly doubled the scores of people without ADN (Mdn = 11), *U* = 867.5, *z* = 2.99, *p* = 0.003. People with ADN had higher emotional dysregulation –seen through the EDS– (Mdn = 134) than those without ADN (Mdn = 88), *U* = 825.5, *z* = 3.24, *p* = 0.001. The tendencies of the two PANAS were opposite. PANAS (+) scores of people with ADN (*M* = 29.76, *SD* = 7.76) were lower than the scores of people without ADN (*M* = 33, *SD* = 7.40), *t*(51.6) = 2.05, *p* = 0.04. However, people with ADN scored higher in the PANAS (−) (*M* = 30, *SD* = 8.46) than people without ADN (*M* = 25.1, *SD* = 7.19), *t*(57.63) = 3.11, *p* = 0.003.

Out of the five NEO-FFI personality factors, the presence of ADN only established a statistically significant difference in Agreeableness. People with ADN (*M* = 43.77, *SD* = 11.22) showed lower Agreeableness in comparison with people without ADN (*M* = 54.83, *SD* = 10.52), *t*(52) = 4.91, *p* < 0.001.

The tests on our traumatic variables yielded no statistically significant results. We found no differences in CTQ component scores and no relationship between ADN and answering the LEC-5.

### 3.2. Logistic regressions

Table 3 summarizes the results of the logistic regression models. Neither the Sociodemographic Vulnerability model, nor the Trauma component of the Psychological Vulnerability model, nor the Health History Risk model had a statistically significant log-likelihood difference from the null model. Apart from the Sociodemographic Vulnerability model, all other models controlled for sociodemographic variables.

**TABLE 3:** Odds ratio and 95% confidence interval of the statistically significant logistic regression models for *ataque de nervios* prediction.

**Regression models**				
	**PV, Anxiety**	**PV, Affect**	**PV, Personality**	**Significant factors**
**Sociodemographic**				
Age	0.97 (0.93, 1.02)	0.99 (0.95, 1.03)	0.98 (0.93, 1.03)	
Gender	0.56 (0.19, 1.68)	0.57 (0.21, 1.51)	0.58 (0.17, 1.97)	
Education	0.14 (0.03, 0.68)**	0.22 (0.05, 0.92)*	0.22 (0.04, 1.33)	0.06 (0.01, 0.46)**
Employment	3.72 (1.05, 13.15)*	1.86 (0.59, 5.83)	2.29 (0.63, 8.40)	2.18 (0.58, 8.17)
Living with partner	1.17 (0.35, 3.95)	1.83 (0.65, 5.12)	6.34 (1.55, 25.97)**	2.39 (0.65, 8.86)
**Anxiety**				
ASI	1.01 (0.97, 1.06)			
BAI	1.06 (1.01, 1.11)*			1.03 (0.99, 1.08)
State anxiety	0.96 (0.88, 1.04)			
Trait anxiety	1.13 (1.06, 1.20)***			1.10 (1.04, 1.17)**
**Affect**				
BDI		1.06 (0.99, 1.13)		
EDS		1.01 (1.00, 1.03)*		
PANAS (+)		1 (0.92, 1.09)		
PANAS (−)		0.98 (0.90, 1.07)		
**Personality**				
NEO-FFI, Neuroticism			1.05 (0.98, 1.12)	
NEO-FFI, Extroversion			1.13 (1.05, 1.21)***	1.12 (1.05, 1.20)***
NEO-FFI, Openness			0.98 (0.93, 1.04)	
NEO-FFI, Agreeableness			0.81 (0.74, 0.88)***	0.88 (0.82, 0.95)***
NEO-FFI, Conscientiousness			1.07 (0.99, 1.15)	

Abbreviations: PV, Psychological Vulnerability; ASI, Anxiety Sensitivity Index; BAI, Beck Anxiety Inventory; BDI, Beck Depression Inventory; EDS, Emotional Dysregulation Scale; PANAS, Positive and Negative Affective Schedule; NEO-FFI, NEO Five-Factor Inventory. * *p* ≤ 0.05, ** *p* ≤ 0.01, *** *p* ≤ 0.001.

In the Psychological Vulnerability model, anxiety severity and the sociodemographic variables of Education and Employment were significantly related to ADN. An education level of college or above was a protective factor against ADN, while the odds of having ADN were 3.72 times higher in employed compared to unemployed people. One-unit increases in BAI and Trait Anxiety scores increased the likelihood of suffering from ADN by 6 and 13%, respectively.

For the affective regulation variables, Education also presented a protective effect. There was a 78% decrease in the odds of having ADN in people with a college or above education level. No affective regulation test appeared as a statistically significant predictor.

After controlling for the NEO-FFI components, the Living with a partner variable, Extraversion, and Agreeableness scores had statistically significant effects on ADN. Individuals with ADN were 6.34 times more likely to live with a partner than those without ADN. Every unit increase in Extraversion increased the odds of suffering from ADN by 13%. The trend for Agreeableness was different; it was a protective factor. Unit increases in Agreeableness were associated with a 19% decrease in the likelihood of suffering from ADN.

The last regression considered all factors with statistically significant *p*-values and odds ratio intervals from the previous three models. The analysis included Education, Employment, the Living with a partner variable, the BAI, Trait Anxiety, Extraversion, and Agreeableness scores. All but Employment, Living with a partner, and BAI significantly predicted ADN. Another protective factor was the Agreeableness score, which decreased the odds by 12% for each one-unit increase. One-unit increases in Trait Anxiety and Extraversion increased ADN odds by 10 and 13%, respectively. An education of college or above reduced the odds of experiencing ADN by 94%. While all other statistically significant variables showed small effect sizes, Education showed large effect sizes in the regressions shown in Table 3.

**FIGURE 2:**
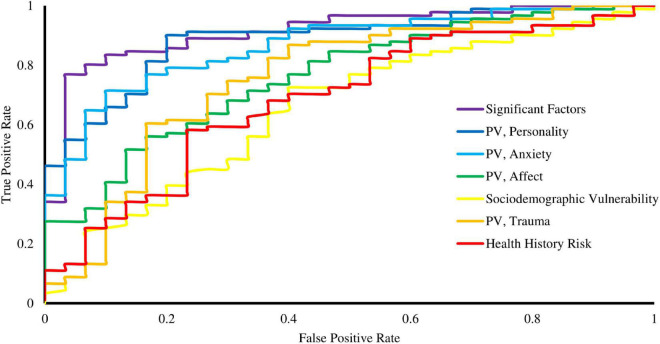
Receiver operating characteristics (ROC) curve of the regression models related to the sociodemographic, health history, and psychological dimensions of *ataque de nervios*. Abbreviation: PV, Psychological Vulnerability.

Table 4 compares the models’ Area Under the Curve (AUC), accuracy, and AIC. Although the Personality category from the Psychological Vulnerability model had the highest accuracy, the Significant Factor model showed the lowest AIC and the largest AUC. Other models lack empirical support to be comparable to this one, considering the differences in AIC are at least 13 points. The Significant Factor model describes the best fit for ADN prediction among our regression models. Figure 2 supports this finding by comparing each model’s receiver operating characteristics (ROC) curve. The curve for the Significant Factors model courses further to the top-left corner of the graph, indicating its superior performance.

**TABLE 4:** Regression model comparison related to the sociodemographic, health history, and psychological dimensions of *ataque de nervios*.

	Significant factors	PV, Personality	PV, Anxiety	PV, Affect	Sociodemographic vulnerability	PV, Trauma	Health history risk
AUC	0.91	0.89	0.87	0.76	0.66	0.76	0.69
Accuracy_cutoff__=__0.5_	85.1%	86.8%	82.6%	78.5%	75.2%	77.7%	76%
AIC	93	106	116	135	143	145	148
ΔAIC		13	23	42	50	52	55

These are ordered according to the difference in AIC with the Significant Factors model. Abbreviation: AIC, Akaike information criterion; PV, Psychological Vulnerability; AUC, Area Under the Curve; ΔAIC, difference in AIC.

## 4. Discussion

Our study highlights the relationships between ADN and certain sociodemographic, health-related, and psychological factors in a Puerto Rican sample of adults from a specialized anxiety clinic. The biopsychosocial model helps clinicians understand how these components interact with one another regarding a particular illness (79). Engel proposed that “by evaluating all the factors contributing to both illness and patienthood, …, a biopsychosocial model would make it possible to explain why some individuals experience an illness which others regard merely as “problems of living,” be their emotional reactions to life circumstances or somatic symptoms” (80). Although we did not evaluate all biological, psychological, and social factors, we did classify the variables we evaluated within similar broad categories.

Our most clinically relevant finding lies in the relationship between education and ADN. According to this study, Puerto Rican adults who completed higher education had reduced odds of experiencing an ADN. This finding supports our hypothesis of ADN being related to “low education.” Therefore, those with an education level of high school or below could be at an increased risk of developing ADN. This is also what Guarnaccia et al. (6) found in their investigation among a Puerto Rican sample aged 17–68 years who lived in a household in Puerto Rico (6). For context, 66% of their sample with ADN had an education of less than high school. However, the results contrast with a more recent study, whose majority of the ADN sample (69%) had an education of 12 years or less, which found no relationship regarding educational level (4). These differences with our results could be due to the stratification of the education variable; we did not separate our sample according to the years studied. Another explanation could be the distribution of education in our sample. The percent of people with an education of college or above in our sample, either with ADN (76%) or overall (79%), is not representative of Puerto Rican adults according to the 2021 American Community Survey 1-year estimates (43%) (81). After considering this statistic, ADN’s prevalence could potentially be underestimated when we factor in the risks we identified in this study and the increasing sociocultural stressors in Puerto Rico. Future studies evaluating the relationship between ADN and educational level would benefit from close attention to the distribution of this variable. Our work is consistent with the literature on how education is a protective factor against psychopathology (82–84). Education’s large effect size generalizes the relevance of addressing educational status in our patients. Disseminating this relationship could help clinicians understand that ADN might be an expression of psychological distress in the context of less formal education. We emphasize the importance of offering psychoeducation and emotional regulation strategies for patients at risk for ADN. For Government officials, these findings stress the need for promoting and developing strategies to increase the population’s access to higher education by recognizing its protective effects on mental health.

Our findings regarding employment were intriguing, especially considering how the literature describes its relation to ADN. Although we hypothesized that ADN would be related to unemployment, to our surprise, the results indicated that those who were employed were at increased risk of having an ADN. Our results contrast previous work which associated being “out of labor force” with an increased risk of experiencing ADN (6). One possibility for this difference may be the amount of employed people in our sample: 67% versus Guarnaccia et al.’s 19% (6). Another reason for this difference may be the increase in workload from 1993 to 2014–2018. It is common knowledge that workload has increased during the past decades. A workload can have variable effects on performance (85, 86), stress levels, decision-making, and hence mental wellbeing (87). It would be interesting to further this analysis by evaluating the association of employment, mental workload, stress, and ADN. Lastly, those with ADN were more likely to live with a partner, which was also the opposite of both our hypothesis and the findings of Guarnaccia et al. (6). They found an increased risk of ADN in women who were widowed, separated, or divorced. It could be possible that those who live with a partner are either experiencing more stress at home or may simply feel more comfortable being vulnerable and expressing their emotions on a full scale. These results suggest that ADN is an important factor regarding interpersonal stress. It is also consistent with previous work that stated ADN expression is naturally social, as 82% of their subjects reported having someone else present during their *ataque* (8). Relationship stress should be more carefully evaluated in patients with ADN. Regardless of the small effect sizes of other variables, future larger-scale studies could clarify their influences on people with ADN. These findings partially support our hypothesis of ADN as a marker of sociodemographic vulnerability, particularly in those with an education of high school or below, employed, and living with a partner.

It is established that ADN, besides being the most consistent predictor of another mental health disorder, is strongly associated with higher rates of psychiatric disorders, particularly anxiety and depression, and psychiatric symptoms (4). Our findings reinforce ADN as a *marker* of more severe anxiety and increased difficulty with emotion regulation (15, 88). This is clinically relevant as ADN could be used to reflect severity in the context of mood and anxiety disorders. Regularly assessing for ADN could help clinicians identify those at-risk patients to tailor their treatment plan. It would be useful to evaluate how ADN patients benefit from either intensive treatment –such as a higher frequency or quantity of psychotherapy sessions– or a combination of psychotherapy and pharmacotherapy.

One of the most interesting findings of our analysis was the high extraversion and low agreeableness in subjects with ADN. This suggests that people with ADN express emotions outwardly and have difficulty accepting situations. This differs from the traditional personality traits in anxiety disorders of high trait anxiety with high neuroticism. However, when thinking about the clinical manifestation of ADN as an episode of intense emotional expression, the presence of high extraversion makes sense. Reactions during ADN episodes –often after an interpersonal stressor– are also consistent with low agreeableness, as this refers to how people tend to behave in their relationships. We believe a closer look at the NEO-FFI scores is valuable for the assessment of our patients’ ADN risk.

It was also interesting that we did not find any relationship between ADN and trauma despite its early clinical descriptions in the literature, where precipitants were described as “obviously traumatic” events (37, 47, 49). More recent studies have not found a direct association between *ataques* and specific psychiatric disorders and results regarding the association of ADN with trauma have been mixed (26). In 2002, the association between childhood trauma and ADN was evaluated in 29 Puerto Rican outpatients yet found no difference (34). Even though Lewis-Fernández et al. (26) suggested that “childhood trauma is a necessary precondition of frequent *ataques*,” they discussed that they were unable to examine the association of trauma exposure due to the very high rate of trauma in their sample (26). However, it is important to note that the association they found refers to an individual experiencing many *ataques* after childhood trauma, rather than at least a single lifetime *ataque*. In the end, the relationship between *ataques* and trauma remains unclear. The lack of a relationship in our sample could be explained by the fact that our data were derived from a clinic where both subjects with and without ADN presented a high prevalence of trauma. It would be interesting to replicate our study by comparing it with subjects who are not actively presenting a mental illness requiring treatment.

We seek to expand on regressions regarding ADN presence. Models can become clinical tools for the early identification and treatment of ADN. Further testing could lead to a set of variables that more accurately reflect the risks for Puerto Rican adults. After this, we can develop a program that predicts the occurrence of an ADN after inputting scores like those in our work. While the increasing quality of the models is evident in Table 3 and Figure 2, studies including a bigger sample and additional mental health screening questionnaires are required for this projection. A model for ADN prediction with an accuracy above 90% seems feasible from our results. However, we first suggest studies that measure whether ADN prevention or ADN treatment provides more benefits to patients’ wellbeing. Our findings guide future prospective studies where risk factors could be used to follow healthy young Puerto Ricans to assess ADN incidence. Another important study on the horizon centers on evaluating the psychometric properties of the expanded CETMA ADN questionnaire that goes beyond the first binomial question and validating it using the current standard questionnaire (89).

An important limitation of our study is that it contained missing values. While we managed the missing data to retain power without adding bias, it would be preferable to double-check questionnaire completion during collection in future works. Our sample was homogenous according to our parameters, however, since our data came from a specialized anxiety clinic, anxiety was probably overrepresented. Because of this, it is important to replicate this study using samples from non-specialized mental health clinics and the general population. It would also be interesting to analyze samples of Puerto Ricans not living in Puerto Rico and evaluate the use of both the ADNQ and NEO-FFI in non-Puerto Rican samples with less formal education. Another projection regards incorporating screening tools for dissociative disorders and intermittent explosive disorder, which relate to ADN (1), but we lacked the questionnaires to add to the Psychological Vulnerability model. Our findings highlight the need for future studies with more robust designs to understand the clinical relevance of the more severe presentation of anxiety and affect regulation shown by our ADN sample.

Overall, our study found translationally relevant relationships and risk factors regarding ADN. In our sample, ADN related to less formal education in the context of high emotional dysregulation in a person with high extraversion and low agreeableness. It seems ADN is identifying people at risk for anxiety (high trait anxiety, high emotional dysregulation, low positive affect, and high negative affect) but, at the same time, a tendency to express emotions outwardly (high extraversion) and having difficulty accepting situations (low agreeableness). As previously stated, these personality traits that differ from those in anxiety disorders point to a distinct profile seen in employed, educated, adult Puerto Ricans living on the Island experiencing anxiety. Cultural differences may explain the dissimilarity in the personality factors associated with anxiety disorders, which lead to variable clinical presentations. This finding can also help clinicians explain to patients with ADN how cultural factors affect their anxiety, as well as their reactions to threats. Given our current findings, a more exhaustive evaluation of ADN is clinically valuable when working with underrepresented research populations. In conclusion, our study amounts to the growing literature on ADN and identifies tools that can be integrated into clinical settings. Through this work we facilitate the comprehension of our patients’ ADN experience, enriching our practice as health providers to deliver better management and care in the most culturally competent manner.

## Data availability statement

The data analyzed in this study is subject to the following licenses/restrictions: The raw data supporting the conclusions of this article are available on request from the corresponding author. Requests to access these datasets should be directed to KM, karen.martinez4@upr.edu.

## Author contributions

AS-V and KM wrote the original research study protocol from which the data was extracted (A3910119). MR-M analyzed the data. All authors contributed to the design, data collection, interpretation of results, writing, and revision of the manuscript.
